# Nutrients handling after bariatric surgery, the role of gastrointestinal adaptation

**DOI:** 10.1007/s40519-021-01194-5

**Published:** 2021-04-24

**Authors:** Stefania Camastra, Maria Palumbo, Ferruccio Santini

**Affiliations:** 1 Department of Clinical and Experimental Medicine, University of Pisa, Via Roma, 67, 56126 Pisa, Italy; 2 Interdepartmental Research Center “Nutraceuticals and Food for Health”, University of Pisa, Pisa, Italy

**Keywords:** Obesity, Bariatric surgery, Nutrients handling, Glucose disposal, Gastrointestinal adaptation, Insulin sensitivity

## Abstract

Bariatric surgery determines a rearrangement of the gastrointestinal tract that influences nutrient handling and plays a role in the metabolic changes observed after surgery. Most of the changes depend on the accelerated gastric emptying observed in Roux-en-Y gastric bypass (RYGB) and, to a lesser extent, in sleeve gastrectomy (SG). The rapid delivery of meal into the jejunum, particularly after RYGB, contributes to the prompt appearance of glucose in peripheral circulation. Glucose increase is the principal determinant of GLP-1 increase with the consequent stimulation of insulin secretion, the latter balanced by a paradoxical glucagon increase that stimulates EGP to prevent hypoglycaemia. Protein digestion and amino acid absorption appear accelerated after RYGB but not after SG. After RYGB, the adaptation of the gut to the new condition participates to the metabolic change. The intestinal transit is delayed, the gut microbioma is changed, the epithelium becomes hypertrophic and increases the expression of glucose transporter and of the number of cell secreting hormones. These changes are not observed after SG. After RYGB—less after SG—bile acids (BA) increase, influencing glucose metabolism probably modulating FXR and TGR5 with an effect on insulin sensitivity. Muscle, hepatic and adipose tissue insulin sensitivity improve, and the gut reinforces the recovery of IS by enhancing glucose uptake and through the effect of the BA. The intestinal changes observed after RYGB result in a light malabsorption of lipid but not of carbohydrate and protein. In conclusion, functional and morphological adaptations of the gut after RYGB and SG activate inter-organs cross-talk that modulates the metabolic changes observed after surgery.

**Level of evidence** Level V, narrative literature review.

## Introduction

Bariatric surgery determines substantial changes in body weight and results in significant improvements or remission of obesity-related metabolic diseases such as type 2 diabetes [1]. The mechanisms underlying these changes have not been completely comprehended. Several factors are involved including endocrine adaptation implicated in energetic metabolism, satiety, changes in brain activity in response to food stimulus and inflammation [2–8]. The gastrointestinal tract is the direct target of bariatric procedures. Thus, it is plausible to suppose that the gastrointestinal rearrangement—accompanied by its functional adaptation to the new anatomical condition and by a consequent change in the kinetics of nutrients absorption—plays a role in the metabolic improvements observed after surgery. The macronutrient’s disposal after a meal varies depending on the anatomical changes after a specific surgical technique, resulting from several mechanisms, such as changes in gastric emptying [9, 10] and gut adaptation [11], changes in entero-hormonal release including incretins [12], changes in nutrients digestion and absorption, [13] and in intestinal microbiota [14]. The identification of the modifications in nutrients disposal after a meal can help understand the metabolic improvement after bariatric surgery.

This review is aimed at summarising the key knowledge around gastrointestinal adaptations after bariatric surgery, including their effect on meal handling and on macronutrients disposal, as well as on insulin sensitivity.

**Fig. 1:**
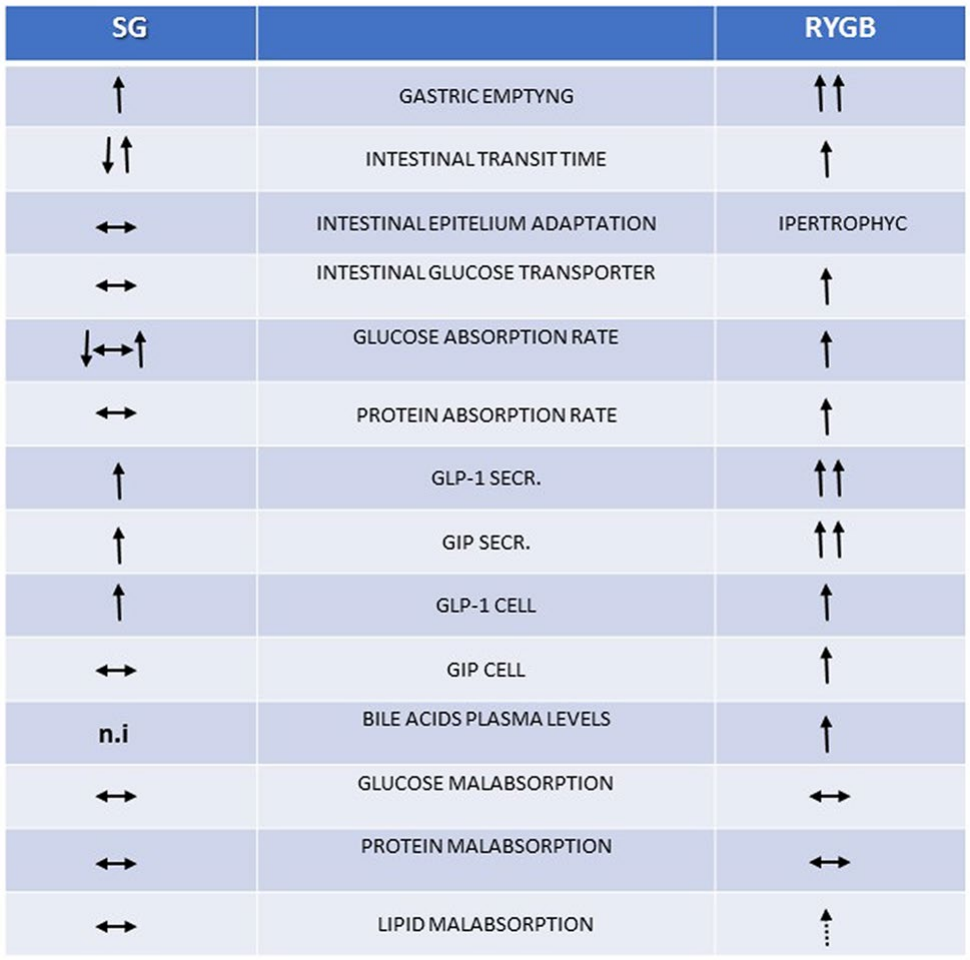
The table summarizes anatomic and functional changes in gastrointestinal tract after RYGB and SG. ↑: increased; ↓: decreased; ↔ unchanged; *n/a*: the information is unavailable or not found

We will compare, where possible, two of the most practiced surgical techniques: Sleeve Gastrectomy (SG) and Roux-en-Y gastric bypass (RYGB). Figure 1 summarizes anatomic and functional changes in gastrointestinal tract after RYGB and SG.

## Gastrointestinal adaptations

### Gastric emptying and intestinal transit

The gastrointestinal tract is the first system to encounter the ingested nutrients. The transit of food through the stomach and intestine has an important role for digestion and absorption of nutrients and for post-prandial metabolism.

The upper intestinal transit, that includes gastric emptying and small intestinal motility, contributes to the regulation of satiety and energy intake. It influences the absorption and the rate of appearance of ingested nutrients in the circulation, the gut hormone signalling and the gut microbiota with a consequent involvement in glycaemic control and insulin sensitivity [15–17].

Modification in gastrointestinal transit may play a role in the aetiology of metabolic diseases [18]. The mechanisms are not clear but the gastric distention, the arrival of nutrient in the small intestine and the release of enteric hormones are involved [19].

All bariatric surgery procedures determine, in different ways, a rearrangement of the gastrointestinal tract. The SG, one of the two procedures analysed in this review, is an example of restrictive surgery consisting of a longitudinal excision of the greater curvature of the stomach, leaving a tube for nutrient passage and preserving the pyloric sphincter. RYGB, the other bariatric surgery procedure herein analysed, determines anatomical rearrangements of the upper gastrointestinal tract with the creation of a small gastric pouch (30 ml) anastomosed directly to the distal portion of the jejunum. This results in the exclusion of a large part of the stomach, of the duodenum and of the upper part of the jejunum to the flow of meal. Bowel continuity is restored via an anastomosis between the Roux limb and the excluded biliopancreatic limb approximately 75–150 cm distal to the gastrojejunostomy. This is considered an example of mixed surgery, restrictive with a malabsorptive component.

Bariatric surgery procedures influence the dynamic of the gastric emptying and, most of them, also the dynamic of the intestinal transit. Gastric emptying, that modulates the delivery of meal to the proximal small intestine, is the principal determinant of the systemic rate of appearance of ingested nutrients. Most of the studies on gastric emptying after SG with radionuclides describe an accelerated gastric emptying for liquid [20, 21] as well as for solids [20–22]. In these studies, the gastric emptying is described as gastric emptying half-time (*T*½) and as percentage of retention of meal after ingestion. Only Bernstine et al. found, in 21 patients who underwent SG with antrum preservation [23], unchanged gastric emptying 3 months after intervention compared to preoperative measures. The contrasting results in gastric emptying measures could be due to the different surgical techniques that use bougies of different sizes and call for a different distance of resection from the pylorus with a consequent different antrum preservation [24].

A recent meta-analysis by Vargas et al. [25] examined times of gastric emptying after different bariatric procedures and its association with weight loss, confirming an accelerated gastric emptying after restrictive procedure. The analysis included nine studies with SG and found that after surgery, the pooled mean reduction in gastric emptying *T*1/2 at 3 months was 29.2 min (95% CI 40.9–17.5 min; *I*^2^ = 91%). Placebo interventions reduced gastric emptying *T*1/2 by 6.3 min (95% CI 10–2.6 min). Changes in gastric emptying were associated with weight loss after SG.

After RYGB, the anastomose of the jejunum to the stomach allows the meal ingested to pass freely to the gut. Studies focusing on the evaluation of gastric emptying after RYGB used direct techniques as scintigraphy [26, 27] or the indirect method by acetaminophen [28] and d-xylose [29] and showed an accelerated gastric emptying after liquid meal ingestion compared to non-operated subjects. The results obtained after a solid meal ingestion were not so clear. Already thirty years ago, using scintigraphic technique, Horowitz et al. [26] studied 12 patients 1 year after RYGB, and observed slower solid emptying and faster liquid emptying after gastric bypass surgery. There was no correlation between stomal size and rates of solid or liquid emptying nor between the weight loss produced by the surgery and the rates of solid or liquid emptying, stoma or pouch size. More recently, Dirksen et al. [27] found a faster gastric emptying both for liquid and for solid in 17 patients studied between 14 and 26 months after surgery compared to non-operated normal weight patients.

A study from the same group [30] compared gastric emptying in patients who underwent RYGB vs patients who underwent SG vs a control group matched for age, BMI and sex. They evaluated gastric/pouch emptying using the time-to-peak of paracetamol concentrations (Tmax pcm). The results showed that the paracetamol absorption was faster in both surgical groups compared with controls, and in RYGB compared to SG, as indicated by a shorter time-to-peak of paracetamol (15 ± 2 vs 36 ± 6 vs 105 ± 19 min; respectively, RYGB vs SG vs controls; *p* < 0.01). The accelerated gastric emptying after RYGB does not appear to be influenced by the macronutrient composition of the meal [31].

Besides gastric emptying, the intestinal transit may be affected by bariatric surgery. The studies that have analysed the intestinal transit after SG by scintigraphy or cine magnetic resonance imaging (MRI) [32–35] found that the bowel transit time was accelerated and the meal reached the terminal ileum faster. Melissas et al. found also that after consumption of a semisolid radiolabeled meal, in 21 patients four months after SG, initiation of cecal filling and the ileocecal valve transit was delayed. The authors suggested that the contact of food with the distal small bowel mucosa had a role in the metabolic effects of SG that occurred before substantial weight los**s** [33].

In patients who underwent RYGB, after the arrival of food into the jejunum, the transit in the small bowel was quicker or unaltered after a liquid meal [36, 37]. The results of the study by Dirksen et al. [27] showed a prolonged intestinal transit both for the liquid and the solid components of a meal during which these were ingested separately but consecutively. The authors speculated that the delayed of small intestinal transit could be a mechanism that limits the malabsorption after RYGB. In the same study, no difference was found in post RYGB and control group regarding the colonic transit, indicating that the mechanisms activated by RYGB do not involve the colon [27].

### Enteroplasticity

Several studies have focused on intestinal adaptation after bariatric surgery. Studies in rats demonstrate that the Roux limb displays hyperplasia and hypertrophy [38, 39] and that the exposition of the Roux limb to undigested nutrients determines an adaptation of intestinal glucose metabolism such as change in glucose transporter and glucose uptake [40, 41].

Cavin et al. [42] confirmed the change in gut morphology not only in rats, but also in humans after RYGB. In rats, they found a hypertrophic alimentary Roux limb (RL) with an increased diameter compared to the biliopancreatic limb or sham operated animals. The villus height and crypt depth were increased in RL and the crypt cells were highly proliferative. In humans, the RL was hypertrophic with an increase in crypt depth and cell proliferation, but there were no changes in villus height.

The intestine is a large organ, responsible for glucose absorption. A change in intestinal morphology after RYGB is associated with a change in intestinal glucose metabolism. A study in humans [43] found in RL an increase of the expression of sodium-glucose transporter 1 (SGLT1), the enzyme responsible for the sodium-dependent active uptake of glucose across the apical membrane of the small intestine. The peak of plasma glucose was related to the expression of SGLT1. However, Baud et al. [44] demonstrated, in a RYGB model of mini pigs, that the intestinal uptake of ingested glucose is blunted in the bile-deprived alimentary limb. Saedi et al. found that in rats, after RYGB, the intestine exhibited the highest rate of glucose uptake. The authors suggested that the increased intestinal glucose uptake should be predominantly mediated through glucose transporter-1 (GLUT1), as suggested by the increase in RNA and protein levels of GLUT1 [40]. More recently, Cavin et al. [42] confirmed that the GLUT1 gene, normally poorly expressed in adult intestine, was overexpressed in the basolateral membrane of hyperplastic RL and not in the biliopancreatic limb after RYGB, both in rats and in humans. Greater amounts of absorbed glucose remained within the RL mucosa. Forty days after surgery, they found an increased expression of SGLT1, GLUT2, and GLUT5 genes in the RL in rats. The authors interpreted these results speculating that whereas the early induction of GLUT1 could sustain the increased bioenergetic demands of intestinal remodelling after RYGB, the overexpression of the others intestinal glucose transporters, could facilitate the increase in sugar absorption to avoid malabsorption [42, 43].

Unlike RYGB, no signs of intestinal hyperplasia and hypertrophy have been observed after SG [42, 45]. Similarly, no change in the expression of glucose transporter was observed in the intestinal tract after SG [42]. Cavin et al. found that the transport of alimentary glucose from the lumen to the serosal side of jejunum both 14 and 40 days after SG in rats was markedly decreased, suggesting a lower absorption capacity of glucose [42].

### Enterohormones

Gastric emptying which controls the rate of appearance of ingested food in the small intestine, intestinal transit and small intestinal nutrient sensing influences the entero-hormones secretion inhibiting ghrelin secretion and stimulating incretin glucagon like peptide-1 (GLP-1), Gastric Inhibitory Polypeptide (GIP), peptide YY (PYY) and cholecystokinin (CCK) secretion during and after meals [5]. Changes in hormone levels lead to gastrointestinal and central nervous system events whose outcome is to inhibit eating [5, 8].

Changes of incretin levels are thought to be involved in the improvement of glucose metabolism after bariatric surgery.

A known characteristic of RYGB and SG, both in animals and in humans, is the postprandial increase in several gut peptides, including the GLP-1 [31, 46], an entero-hormone secreted from intestinal L-cells that stimulates insulin secretion and decreases glucagon production. Administration of exogenous GLP-1 or GLP-1 analogues results in weight loss and improvements in glucose regulation both in type 2 diabetic and non-diabetic patients [47, 48].

The incretin secretion could be also influenced by the gut adaptation. As an effect of hypertrophy, 14 days after RYGB, an increase of GLP-1 and GIP secreting cell has been described in the jejunum mucosa of Roux limb in rats. No change was observed in cell density [42]. Conflicting results were observed in GLP-1 and GIP secreting cell density in humans, this being found either unchanged [42] or increased in Roux limb more than 4 months after surgery [49, 50].

Although SG does not induce hypertrophy, it determines an increase of the number and density of GLP1-secreting cells, and not of GIP-secreting cells, coincidental with the plasma increase of GLP-1 after an oral glucose load. [42].

These results could indicate a role of entero-plasticity on the gut hormones modifications and of the metabolic effect of bariatric surgery.

### Bile acids

Several studies indicate that bariatric surgery leads to an increase in plasma levels of bile acids (BA) [51–56]. After RYGB, an increase was found in fasting and postprandial BA, with parallel alterations in composition. A more modest increase in both fasting and postprandial BA was observed after SG, but the results were not univocal [31, 57].

Despite clear evidence for an increase in plasma BA after bariatric surgery, the cause is unknown. It could include increased hepatic synthesis or altered enterohepatic recirculation of bile. In a study from our research group, we found that after RYGB, the patients had high plasma BA but normal levels of BA synthesis markers [51]. This may indicate that an altered BA transport could be responsible for high plasma BA. The two major determinants of BA transport are absorption from the intestine and uptake into the liver. It may be possible that after RYGB, uptake of BA in the small intestine is enhanced. This would be consistent with the increase in FGF19 observed in this and others studies after RYGB [51, 55, 58] and with the increased postprandial BA excursions that occur after RYGB [59]. This may also explain the preferential increase of conjugated BA observed after RYGB.

It has been proposed that increased BA may contribute to the metabolic improvements after surgery [54, 60, 61]. Some evidence demonstrates that BA improves glucose metabolism, through the modulation of the nuclear receptor farnesoid X receptor (FXR) and the cell surface receptor G protein-coupled bile acid receptor 1 (TGR5) [62, 63]. In the intestine, the activation of TGR-5 by endocrine L cells leads to increased secretion of GLP-1 that increases insulin secretion by pancreatic β-cells and inhibits the secretion of glucagon by pancreatic α-cells [64–66]. Additional evidence indicates [67] that GLP-1 secretion by intestinal L cells is negatively regulated by FXR through inhibition of pro-glucagon gene expression and suppression of GLP-1 secretion. These results suggest that BA activation of TGR5 and FXR in intestinal L cells induces opposite effects on GLP-1 secretion. Shapiro et al. hypothesized [68] that after food ingestion, TGR5 activation in L cells probably occurs rapidly, whereas activation of FXR induces a more delayed response. This difference leads to a temporal separation between postprandial positive effects of BA-TGR5 signal on GLP-1 secretion and FXR-mediated inhibition of GLP-1 release [67], with a net effect of improvement of glucose metabolism.

Other possible mechanisms to explain the positive effect of elevated BA following bariatric surgery are the direct activation of FXR in the liver, which leads to reduced gluconeogenesis [64] and the activation of FXR in muscles, liver and adipose tissue which leads to improvements in insulin sensitivity [69]. However, the increase in fasting plasma BA occurs relatively late after surgery, and therefore it cannot explain the early improvements in glycemia after surgery [70].

Changes in gut morphology following bariatric surgery together with diet modification alter intestinal gut microbiota, with differences depending on the technical surgery adopted. [71, 72].

A study in which faeces from RYGB-treated patients was transplanted to germ-free mice, resulted in a significant greater fat loss in recipient mice, suggesting that the altered microbiome per se contributes to weight loss [73].

Furthermore, gut microbiota is a key regulator of BA conjugation and secondary BA formation [74] with differing affinity for FXR or TGR5 and thus different metabolic effects [75, 76].

However, although the microbiota seems to have a role in metabolic regulation, due to the different results of the studies that analyse the change in microbiota after surgery [73, 77–81], it is unclear if its role can be crucial for the metabolic effect of bariatric surgery.

### Nutrient handling

The macronutrient disposal may differ among various surgical techniques, depending on their impact on meal size and relatively to malabsorptive effects.

### Carbohydrates

Several studies, have evaluated the impact of RYGB and SG on carbohydrate disposal after a meal, using a mixed meal test combined with stable isotope-labeled tracers in conjunction with mathematical modeling [30, 46, 82]. The carbohydrate component of the meal consisted in a liquid glucose solution. The results of these studies indicate that, both early [82] and long after surgery [46], the post-meal plasma glucose and insulin profiles are extremely altered by the surgery. In a study of our group [46], a large excursion of glucose was observed one year after RYGB. The peak of blood glucose was observed at 60 min after the meal ingestion, followed by a rapid drop below the basal levels. The time course of insulin secretion reproduced the time course of plasma glucose*.* The study compared patients with diabetes to those without diabetes before surgery. In both groups, the tracer analysis showed a temporal pattern of oral glucose appearance in the circulation similar to the glycaemic and insulinemic pattern, but very different compared to pre-surgery and to a non-operated BMI-matched group. After surgery, during the first hour following the ingestion of 75 g of glucose, approximately 35 g appears in the circulation, corresponding to 45% of the total amount ingested compared to about 30% of the pre-surgery condition.

Compared to pre-surgery condition, the amount of glucose appearing in the circulation in the first hour after the meal ingestion increased of 30% in non-diabetic and 20% in diabetic patients, corresponding to 70% and 60% of the glucose appeared in the circulation in the 5 h of the study, respectively, for non-diabetic and diabetic patients [46, 82]. These proportions were in line with the results of other studies that used similar tracer techniques [83, 84].

Although the surgery may change in a striking way the time course of glucose appearance in the circulation, only a slight reduction is observed in the total amount of glucose absorbed, during the 5-h post-meal ingestion. In our study, we observed a reduction of 6% in non-diabetic patients and 3% in diabetic patient compared to pre-surgery [46]. Other studies confirmed a reduction of about 4% of the total oral glucose appearance after a mixed meal compared to pre-surgery [84].

A recent study [30] using stable isotopes, compared post-prandial glucose absorption and metabolism in non-diabetic patients after RYGB with patients who had undergone SG and with non-operated patients. The authors confirmed the previous evidences of rapid post-prandial glucose uptake after RYGB that they found, to a lesser extent, even after SG. The peak of the rate of appearance of glucose after RYGB was 33% higher compared to SG. The overall 6-h post-meal recovery of glucose ingested was similar in post RYGB, post SG and the control group, but the recovery observed in the first hour after meal ingestion was higher after RYGB followed by SG and the control group (31% vs 23% vs 14% of ingested glucose, p 0.001 vs RYGB).

**Fig. 2:**
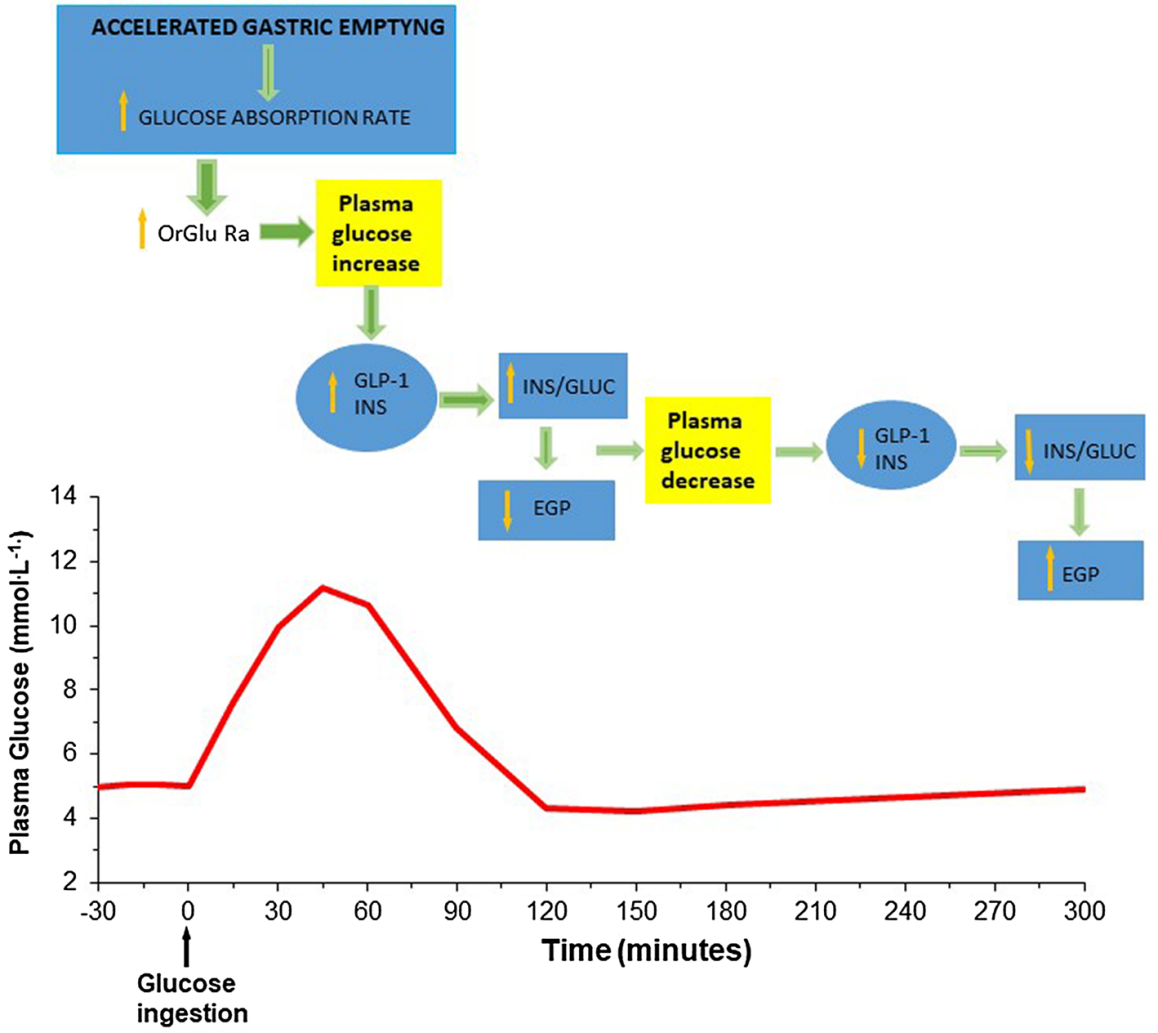
Principal determinants of postmeal plasma glucose profile after RYGB. *OrGlu-Ra* rate of oral glucose appearance, *GLP-1* glucagon like peptide-1, *INS* plasma insulin, *INS/GLUC* molar ratio from insulin secretion values and plasma glucagon during the postmeal period, *EGP* endogenous glucose production. The red line in the lower part of the figure describes the temporal pattern of plasma glucose after glucose ingestion in patients after RYGB. In the upper part of the figure are described the principal determinants of the post meal glucose excursion. The temporal pattern of post meal plasma glucose after RYGB is mainly the result of the altered delivery of the ingested glucose into the systemic circulation, due to an accelerated gastric emptying and increased glucose absorption. The accelerated gastric emptying causes an abnormal increase in the GLP-1 release in response to a meal which contributes to increased insulin secretion. The consequent increase in the insulin to glucagon molar ratio (INS/GLUC) determines the suppression of EGP with the consequent tendency to hypoglycemia observed 2 h after glucose ingestion. After the peak at the first hour after the meal, the INS/GLUC drops rapidly in phase with the reduction of plasma glucose with a consequent increase of EGP that is critical to prevent post prandial hypoglycaemia

From these studies, we can conclude that the observed pattern of plasma glucose after meal stems from altered glucose delivery in the systemic circulation as a result of accelerated gastric emptying (Fig. 2), in line with the observation that plasma glucose concentration increases with the increase of the rate of gastric emptying [85]. The entero-plasticity with the adaptation of intestinal epithelium and the change in the glucose transporter density facilitates the phenomena and accounts for the unchanged co-efficient of carbohydrate absorption found after RYGB [13] and for the absence of carbohydrate malabsorption [29].

The accelerated gastric emptying after RYGB and SG determines an increase of GLP-1 release from L-Cell in response to a meal [12, 46], which contributes to the stimulation of the pancreatic beta cell to increase insulin secretion with the consequent tendency to hypoglycaemia observed 2 h after glucose ingestion mostly in patients who underwent RYGB [46] (Fig. 2). Not only GLP-1 but also other gastrointestinal hormones, such as GIP, PYY and CCK, result in the increase in both after RYGB and SG, although the peak concentration is much higher after RYGB than SG [30, 46]. Ghrelin is an exception, being reduced after both procedures, especially after SG [30]. The larger increase in glucose and hormones after RYGB compared to SG, and to non-operated patients, is mainly related to the more accelerated gastric emptying observed after RYGB. Other factors that may contribute are the direct delivery of nutrient to distal gut and the more pronounced, or exclusive, modification observed after RYGB compared to SG, which is related to the entero-plasticity described above (hyperplasia of gut epithelium that results in increased glucose transporters and glucose uptake, increased enteroendocrine cell, changes in BA, changes in microbiota). The post-meal response of gut hormone depends on the macronutrient ingested. Carbohydrates are, in fact, almost exclusively responsible for the significant post-meal increase of GLP1 after RYGB, as well as for the increase of GIP compared to fat and protein meal [31]. The almost exclusive response of GLP1 and GIP to carbohydrate consolidates the possibility of their precocious absorption in the Roux limb, as indicated by increased glucose transporters and enteroendocrine cell expression due to gut adaptation.

In our above-mentioned study [46], we observed that, in synergy with glucose absorption, the time course of endogenous glucose production (EGP) after meal ingestion showed an initial suppression 1 year after RYGB. This was followed by a recovery from the second hour after the meal, when plasma glucose concentrations tended to the basal value or below [46] (Fig. 2). Similar results were reported by other authors after RYGB but not after SG; in fact, after the latter, the post-prandial time course of EGP was similar to pre-surgery state [86] and to the control group [30]. The response of EGP to the meal is critical to balance the excessive postprandial glucose concentrations early after meal ingestion, and to prevent hypoglycaemia later when plasma glucose declines. The post-meal EGP is probably a consequence of the balance of the insulin suppression and glucagon stimulation on hepatic glucose production. We can assume this from the rapid increase of plasma glucagon after the meal, which remained above basal levels throughout the absorption period [31, 46]. Consequently, the pre-hepatic insulin-to-glucagon ratio, dropped rapidly after the first hour peak in phase with the lower plasma glucose levels [46] (Fig. 2).

### Proteins

In normal condition, although protein absorption mainly takes place in the proximal jejunum, the entire small bowel can perform this function through the action of the enzymes from exocrine pancreas and the proteases located in the brush-border membrane of the enterocytes [87]. Despite the exclusion of the majority of the stomach, and the alteration in the mixing of gastro-pancreatic secretions in the distal part of the jejunum, RYGB does not seem to cause protein malabsorption.

After long-limb RYGB (a variant of RYGB in which the excluded limb is lengthened), the coefficient of protein absorption, calculated from the nutrient intake and faecal output, resulted as non-significantly reduced [13, 88].

A study in rat using a sensitive 156N meal test, showed that RYGB improved protein digestibility compared to SG or sham operated. Fractional protein synthesis was scarcely altered by bariatric surgery. Compared to sham, the 15 N distribution was retained in the re-modeled mucosa of RYGB; however, protein retention in the liver and muscles was lower, probably due to a higher uptake of dietary nitrogen by the hypertrophic intestinal mucosa [89].

Results in humans are only partially consistent. A study on post-meal protein handling, conducted three months after RYGB, found an accelerated appearance of amino acids derived from ingested protein. This study used a semi-liquid mixed meal containing intrinsically labelled calcium caseinate as protein source, combined with labelled leucine infusion [90]. Neither the alteration of gastric acid secretion [91] or the delay of the mixing of the pancreatic juice with the meal seem to influence protein digestion [92] after RYGB. Since the pancreatic enzymes are crucial for protein absorption, it is reasonable to assume that the accelerated protein absorption after RYGB reflects a rapid release of nutrients from the upper limb anastomosis, as observed after a liquid meal [36].

As compared with RYGB, protein handling after SG has not been extensively studied. In a recent study, Svane et al. [30] compared SG- and RYGB-operated subjects matched for weight loss and in the weight-stable phase. Using a semi-liquid mixed meal containing intrinsically labelled protein source combined with labelled amino acid infusion, they found a kinetic of absorption markedly different in RYGB compared to SG. After SG, patients showed a rate of systemic appearance of phenylalanine from ingested protein similar to controls, except for an earlier peak. On the contrary, the peak of circulating phenylalanine was higher in RYGB patients, suggesting an accelerated protein digestion and absorption rates only after RYGB. Despite the different absorption kinetic, during the 6-h study, the post-prandial recovery of the ingested proteins was comparable between the groups. These results indicate that there are no changes in the splanchnic extraction of amino acids and there is no protein malabsorption both after RYGB and SG. Furthermore, the study suggests that the accelerated protein digestion and absorption rates persist long after RYGB, when patients are weight-stable.

Accelerated protein digestion and amino acid absorption can influence the metabolic and hormonal changes that occur after RYGB. Amino acids are able to stimulate insulin secretion from pancreatic beta cell [93, 94] and GLP-1 secretion from intestine L-cell [95]. After RYGB, the accelerated protein digestion that follows a meal may enhance post-prandial insulin secretion and GLP-1 release [82, 83] even if it is less than the carbohydrate component of the meal [31]. Furthermore, the protein component of the meal seems to stimulate CCK secretion after RYGB more than the fat component [31].

Amino acids represent a major stimulus to glucagon secretion independently from surgery as indicated from a similar increase in non-operated patients after protein ingestion [31] and from an increase of glucagon also after intravenous administration of amino acids [96].

### Lipids

Under normal conditions, lipids enter into the duodenum eliciting the release of CCK by enteroendocrine cells. CCK stimulates small intestinal motility and the release of bile and pancreatic lipases. After digestion, monoglycerides and fatty acids are transported into jejunal enterocytes, combined with apolipoproteins B48 (ApoB48) and other apolipoproteins to be released as chylomicrons.

Following RYGB, lipids do not pass through the duodenum and consequently the secretion of bile and pancreatic enzymes is reduced [37]. The reduction of exocrine pancreatic function has been found prevalently in patients undergoing distal RYGB in which a shorter common limb was performed [97].

The delayed dietary fats transformation and the delayed formation of micelles could limit the amount of fat available for absorption. Several studies have found an increase of faecal fat excretion following RYGB compared to non-operated patients or to patients that underwent restrictive surgery though not as high as after malabsorptive surgery [13, 37, 98]. A study conducted on patients that underwent long-limb RYGB [13] calculated that the coefficients of fat absorption averaged 92.1% before surgery and decreased averaging 71.9% at 5 months and 68.1% at 14 months after surgery. However, the amount of calorie loss did not seem to be a major contributor to weight loss in comparison with the reduction in food intake. More recently, Moreland et al. [88], confirmed a reduction of average fat absorption co-efficient by 20.3% from pre-bypass levels one year after RYGB. The reduction of fat absorption could explain the reduction of post-meal triglycerides concentration after mixed meal found after RYGB compared to pre-surgery [99, 100].

A study [101] conducted on a small group of patients proposes a different mechanism to explain the triglycerides reduction after RYGB. Compared to non-operated patients, after a mixed meal ingestion, the RYGB patients showed an earlier but smaller peak of triglyceride, an earlier peak and higher values of BA and CCK concentration, and a higher, although not significant, peak of ApoB48. ApoB48 is the primary protein component of chylomicrons and its production depends primarily on the amount of fat absorbed. Thus, the authors interpreted the combination of a lower postprandial triglycerides peak with the early postprandial ApoB48, and bile acid response as a consequence of an accelerated delivery of lipids to distal small intestine with a consequent enhanced fat absorption and an increased metabolic clearance of plasma triglycerides.

From animal studies emerges, both after RYGB and SG, a normal absorption of intestinal lipids but a reduction of triglycerides secretion in the circulation after lipid ingestion [102, 103]. Thus, an improvement of postprandial lipid clearance has been thought to be a possible mechanism of reduction of post-meal triglycerides concentration observed after SG and RYGB [100, 104].

### Insulin sensitivity

An important consequence of weight loss after bariatric surgery is the improvement of tissue insulin sensitivity [105] with a more efficient peripheral utilisation of glucose that contributes to the improvement of glycaemic control especially in diabetic patients. The effect of bariatric surgery on insulin sensitivity has been evaluated in a large number of studies that used different methods to assess insulin action.

Unlike the predominantly malabsorptive surgery, such as biliopancreatic diversion, in which the insulin sensitivity is completely restored both in diabetic and non-diabetic patients [106–108] early after surgery and independently from weight loss, most of the studies using euglycaemic hyper-insulinemic clamp, the gold standard method to measure peripheral insulin sensitivity, indicate that in humans, the recovery of insulin sensitivity after RYGB [82, 105, 109] or SG [86, 110], is proportional to the weight lost.

By combining euglycaemic hyperinsulinemic clamp with double-tracer infusion, it is possible to evaluate tissue insulin sensitivity in humans [105]. With this combined technique, we observed early after RYGB an improvement in hepatic insulin sensitivity, as indicated from an unchanged EGP in the context of lower insulin levels. This result can be explained by the calory restriction that patients experience early after RYGB, as indicated also by the significant fall in basal energy expenditure and by an increased lipolysis with an increase of lipid oxidation whereby lipids replaced carbohydrate as oxidative fuel [105]. Muscle insulin sensitivity early after surgery remained unchanged [105, 111].

Long after RYGB, a significant improvement of hepatic, adipose tissue and muscle insulin sensitivity can be observed. These elements are not completely normalised compared to lean controls, but they are proportionate to weight loss [105, 111].

Insulin resistance in obese patients is related to ectopic fat accumulation, inflammation and change in adipose tissue histology characterized by adipocyte hypertrophy, degenerative changes and necrosis, macrophage infiltration and formation of crown-like/cyst-like structures. [112, 113].

The improvement in insulin sensitivity after bariatric surgery is related to an improvement in plasma inflammatory biomarkers [112], as well as to a reduction in liver [114, 115] and muscle fat accumulation [112, 116], and to changes in subcutaneous adipose tissue histology with a consequent reduction of the adipocytes area and of the peri-adipocytes inflammation [112]. However, despite the large weight loss observed after surgery, insulin sensitivity does not completely normalize and sparse macrophages (mainly in T2D patients) are still present in SAT [112, 117].

Gut adaptation after surgery and the consequent reprogramming of intestinal glucose metabolism, could make the gut an important organ for glucose disposal, contributing to the improvement in glycaemic control after RYGB through insulin-dependent and insulin-independent mechanisms. Intestine is highly responsive to insulin in animals and humans, as observed under hyperinsulinemic clamp condition, when the intestinal glucose uptake increases of 2.5-fold. Given its large mass, reduced intestinal glucose uptake may impact the whole-body metabolism. Obese subjects show intestinal insulin resistance in post-prandial condition [118].

A lack of suppression of gluconeogenesis or impaired stimulation of glucose transport and metabolism by insulin may both be involved.

At a cellular level, insulin-resistant enterocytes fail to inhibit gluconeogenesis and glucose release from both apical and basolateral surfaces influencing the glucose transporter GLUT2 internalization [119] and resulting in decreased net glucose uptake. Using [18F]fluoro-2-deoxyglucose and positron emission tomography during hyperinsulinaemia, insulin-stimulated intestinal glucose uptake was found increased in non-diabetic patients after RYGB and SG [41]. This result could suggest an improved response of GLUT2 to insulin action, in accordance with the increased expression of glucose transporter genes found in the RL of rats [42].

Another mechanism by which gut could participate to the improvement in insulin sensitivity after surgery could be the one mediated by the increase in bile acids related to peripheral insulin sensitivity [51]. This mechanism has been previously mentioned in this review and could involve the effect of BA in the modulation of TGR5 and FXR, GLP1, gut microbiota [69].

## Conclusion

Bariatric surgery determines a rearrangement of the gastrointestinal tract that influences nutrient handling and plays a role in the metabolic changes observed after surgery. Most of the changes depend from an accelerated gastric emptying observed in RYGB and, to a lesser extent, in SG. This accelerated gastric emptying determines a rapid appearance of glucose in the circulation and a stimulation of entero-hormones with a consequent stimulation of insulin secretion. Protein digestion and amino acid absorption appear to accelerate after RYGB but not after SG. The intestinal changes observed after RYGB probably limit the malabsorption only to a light malabsorption of lipids but not carbohydrates and proteins. Overall, the functional and morphological adaptations of the gut after RYGB and SG activate inter-organs cross-talk that modulates the metabolic changes and influences insulin secretion and insulin sensitivity.

### What is already known on this subject?

The rearrangement of the gastrointestinal tract that occurs after bariatric surgery influences the nutrient handling and plays a role in the metabolic and hormonal changes observed after surgery. The information on the subject in the literature is large but scattered.

### What does this study add?

The review summarises the key knowledge around gastrointestinal adaptations after bariatric surgery and their effect on meal handling, macronutrients disposal, hormonal changes as well as on insulin sensitivity. Overall, the review highlights how functional and morphological adaptations of the gut after RYGB and SG activate inter-organs cross-talk that modulates the metabolic changes and influences insulin secretion and insulin sensitivity.

## Abbreviations

ApoB48: Apolipoproteins B48
BA: Bile acids
CCK: Cholecystokinin
EGP: Endogenous glucose production
FXR: Farnesoid X receptor
GIP: Gastric Inhibitory Polypeptide
GLP-1: Glucagonlike peptide-1
GLUT: Glucose transporter
PYY: Peptide YY
RL: Roux limb
RYGB: Roux-en-Y gastric bypass
SG: Sleeve gastrectomy
SGLT1: Sodium–glucose transporter 1
TGR5: G protein-coupled bile acid receptor 1
